# ANCA associated vasculitis in patients from Saudi Arabia

**DOI:** 10.12669/pjms.341.13881

**Published:** 2018

**Authors:** Abdurhman Saud Al Arfaj, Najma Khalil

**Affiliations:** 1 Prof. Abdurhman Saud Al Arfaj, FRCP©, FACP, Division of Rheumatology, Department of Medicine, College of Medicine, King Saud University, Riyadh, Saudi Arabia; 2 Najma Khalil, M.Sc., M.Phil, Division of Rheumatology, College of Medicine Research Center, College of Medicine, King Saud University, Riyadh, Saudi Arabia

**Keywords:** ANCA associated vasculitis, Antineutrophil cytoplasmic antibodies (ANCA), Eosinophilic granulomatosis with polyangiitis, Granulomatosis with polyangiitis, Microscopic polyangiitis

## Abstract

**Objective:**

To explore clinical and laboratory features, therapy and outcome of antineutrophil cytoplasmic antibodies (ANCA) associated vasculitis (AAV) patients from our tertiary care center.

**Methods:**

This study of AAV patients seen in Rheumatology clinics at King Khalid University hospital (KKUH), King Saud University, Riyadh during the period 1990-2014 was carried out retrospectively. Demographic, clinical, haematological and immunological parameters along with therapy, complications and outcome were retrieved from patients' medical charts. Different characteristics were compared between the three groups of AAV; GPA (Granulomatosis with polyangiitis), MPA (Microscopic polyangiitis) and EGPA (Eosinophilic granulomatosis with polyangiitis).

**Results:**

We identified 34 AAV patients (21 males: 13 females; 31 Saudis: 3 non-Saudis) comprising of 23 GPA, 2 MPA and 9 EGPA cases. The mean age of onset was 42.1±17.6 years (range 11-75) and mean duration of disease was 8.7± 5.1 years (range 1-20). The most frequently affected system was pulmonary in all AAV (73.5%), GPA (65.2%) and EGPA (100%) while it was renal in MPA (100%) patients. Ophthalmological and upper airways involvement was higher in GPA. Neurological involvement was higher in EGPA (p<0.05). ANCA were detected in 79.4% of AAV patients, of them c-ANCA were 77.8% and p-ANCA 22.2%. ANCA was positive in 91.3% GPA, 100% MPA and 44.4% EGPA patients. In GPA c-ANCA were detected in 80.9% and p-ANCA in 17.4%, in MPA, c-ANCA were detected in 50% and p-ANCA in 50%, in EGPA, c-ANCA were observed in 75% and p-ANCA in 25%. GPA patients had PR3 specificity in 93.3%, and MPO in 6.7%, PR3 was present in all MPA patients (100%), while EGPA patients had MPO (100%). Therapy administered were corticosteroids (100%), intravenous cyclophosphamide (58.8%), azathioprine (50%) and rituximab (11.8%). Infections were noted in 29.4%.

**Conclusions:**

The 10-year survival in our AAV patients was 95%. ANCA pattern was similar to Caucasian AAV patients and different from Japanese and Chinese AAV patients.

## INTRODUCTION

Antineutrophil cytoplasmic antibodies (ANCA) associated vasculitis (AAV) is a group of vasculitis disorders characterized by the presence of ANCA which comprise of three diseases; granulomatosis with polyangiitis (GPA) (previously known as Wegener's granulomatosis) (WG), microscopic polyangiitis (MPA) and eosinophilic granulomatosis with polyangiitis (EGPA) (previously known as Churg-Strauss syndrome) (CSS) which are characterized by necrotizing inflammation of small vessels.^1^,^2^ They are differentiated by pattern of ANCA, clinical manifestations and biopsy of involved organ.^3^ ANCA are found in approximately 90% GPA patients, 70% of MPA and 45% of EGPA patients.^4^ Cytoplasmic ANCA (c-ANCA) directed against proteinase 3 (PR3) are mostly detected in GPA whereas perinuclear ANCA (p-ANCA) directed against myeloperoxidase (MPO) are mostly found in MPA and EGPA.^4^,^5^ The upper and lower respiratory tract and the kidneys are the main targets in GPA whereas kidneys are the most commonly affected organs in MPA and pulmonary hemorrhage can also occur.^6^-^8^ EGPA involves three phases comprising of allergic rhinitis and asthma, eosinophilia and pulmonary infiltrates and vasculitis phase of granulomatous inflammation.^9^ Without therapy, the prognosis of AAV is poor, up to 90% dying within two years of disease onset.^6^ Combination therapy with intravenous cyclophosphamide (IV CYC) and high dose corticosteroid has shown to achieve remission in up to 90% patients and has transformed AAV from a fatal disease to a chronic relapsing condition in recent decades.^10^,^11^

AAV has not been studied in patients from our region. This study was undertaken to study the clinical, hematological and immunological features, treatment and outcome of AAV in our patients from Saudi Arabia.

## METHODS

Retrospective chart review of AAV patients followed up in Rheumatology clinics at King Khalid University hospital (KKUH), King Saud University, Riyadh during the period 1990-2014 was performed. The study was approved by Institutional Review Board of College of Medicine and KKUH. We retrieved demographic, clinical and laboratory data of AAV patients including GPA, MPA and EGPA at presentation and follow up. They were diagnosed as per Chapel Hill Consensus Conference (CHCC) definition of GPA, MPA and EGPA.^1^ Clinical information collected included constitutional, opthalmological, ENT, joints, cutaneous, pulmonary, cardiac, renal, neurological and gastrointestinal manifestations. The laboratory profile retrieved included WBC, hemoglobin, platelets count, ESR, Rheumatoid factor, serum creatinine, urine protein, 24 hour urine protein excretion, creatinine clearance and ANCA. ANCA pattern and specificity were also noted, namely c-ANCA, p-ANCA, PR3-ANCA and MPO-ANCA. The method for testing ANCA was indirect immunofluorescence (for detecting c-ANCA and p-ANCA) and enzyme-linked immunosorbent assay (ELISA; for detecting PR3- and /or MPO-ANCA). Modalities of therapy, complications encountered and disease outcome were also recorded. Patients were followed up every three months. They were treated with corticosteroids and a combination of corticosteroids and IV CYC. Treatment also involved initial phase of induction therapy with 1g/day of IV methyl prednisolone for three days. IV CYC induction comprised of administration of 6 doses over 12 months and 12 doses over 24 months period. Azathioprine and methotrexate were prescribed as maintenance therapy. Plasmapheresis and rituximab were administered to control refractory disease symptoms. Statistical analysis of the data was performed using SPSS version 15 and presented as percentages and means. The differences between GPA, MPA and EGPA were compared using chi-square test (categorical variables) and ANOVA (quantitative variables). A p value <0.05 was considered significant. Kaplan-Meier survival analysis was used to compute survival rates and plot the survival curve.

## RESULTS

### Demographic features of whole AAV group

Only 34 cases satisfied diagnosis of AAV who presented to the clinics during 1990-2014. Of them 23 (67.6%) had GPA, 2 (5.9%) had MPA and 9 (26.5%) had EGPA. They included 31 Saudis, 1 Palestinian, 1 Sudanese and 1 Pakistani. Twenty one (61.8%) were males and 13 (38.2%) were females (M: F, 1.6:1). Mean present age ± SD was 47.6 ± 18.8 (range 16-83) years and mean age at onset of disease was 42.1 ± 17.6 (range 11-75) years. Mean interval between onset of symptoms and diagnosis was 5.2 ± 9.3 (range 0-30) months, mean duration of disease was 8.7 ± 5.1 (range 1-20) years and mean duration of follow up was 5.2±4.7 (range 0.1-19) years.

### Demographic features compared in three AAV diseases

When demographic parameters were compared among the three AAV diseases, we found difference between mean age of disease onset between GPA, EGPA and MPA patients. Mean age of disease onset was lower in GPA and EGPA patients (42.1±14.5, range 11-63 years and 34.2± 25.3, range 11-75 years respectively) while it was higher in MPA patients (62.0 ± 12.7, range 53-71 years) though not significant (p>0.05). The M:F ratio in GPA was 14:9, in MPA 2:0 and in EGPA 5:4.

### Clinical features of whole AAV group

Clinical features are presented in Table-I. The most frequently affected system was pulmonary noted in 25 of 34 AAV (73.5%) patients compared to other systems, the next being kidneys, which were affected in 16 (47.1%) patients and also upper airways and cardiac system (both the systems 47.1% each). Arthralgia was also a presenting symptom in substantial number of AAV patients.

**Table-I:** Presenting symptoms in 34 ANCA associated vasculitis patients.

Symptoms	GPA n=23 N(%)	MPA n=2 N (%)	EGPA n=9 N (%)	All AAV n=34 N(%)
*Constitutional:*	12(52.2)	1(50.0)	5(55.6)	18(52.9)
Fatigue	5(21.7)	1(50.0)	2(22.2)	8(23.5)
Fever *	13(56.5)	0(0)	1(11.1)	14(41.2)
Myalgia	4(17.4)	1(50.0)	2(22.2)	7(20.6)
Night sweats	3(13.0)	0(0)	0(0)	3(8.8)
Anorexia	5(21.7)	0(0)	2(22.2)	7(20.6)
Weight loss	8(34.8)	0(0)	3(33.3)	11(32.4)
*Ophthalmological*	9(39.1)	0(0)	1(11.1)	10(29.4)
*Ear*	3(13.0)	0(0)	0(0)	3(8.8)
*Nose and sinus*	13(56.5)	1(50.0)	2(22.2)	16(47.1)
*Oral*	3(13.0)	0(0)	1(11.1)	4(11.8)
*Joints:*	9(39.1)	1(50.0)	5(55.6)	15(44.1)
Arthralgia	9(39.1)	1(50.0)	5(55.6)	15(44.1)
Arthritis	2(8.7)	1(50.0)	0(0)	3(8.8)
*Cutaneous:*	7(30.4)	0(0)	2(22.2)	9(26.5)
Scaly papular	1(2.9)	0(0)	1(11.1)	2(5.9)
Erythematous macular	1(2.9)	0(0)	1(11.1)	2(5.9)
Palpable purpura	3(13.0)	0(0)	0(0)	3(8.8)
Nodular ulcers	2(8.7)	0(0)	0(0)	2(5.9)
*Pulmonary:*	15(65.2)	1(50.0)	9(100.0)	25(73.5)
Asthma*	3(13.0)	0(0)	9(100.0)	12(35.3)
*Cardiac*	12(52.2)	1(50.0)	3(33.3)	16(47.1)
*Renal*	11(47.8)	2(100.0)	3(33.3)	16(47.1)
Neurological*	2(8.7)	0(0)	4(44.4)	6(17.6)
*Gastrointestinal*	6(26.1)	0(0)	4(44.4)	10(29.4)

ANCA: Anti-neutrophil cytoplasmic antibodies, AAV-ANCA associated vasculitis, GPA: Granulomatosis with polyangiitis, MPA: Microscopic polyangiitis, EGPA: Eosinophilic granulomatosis with polyangiitis,

* p <0.05 (significant).

### Clinical features compared in three AAV diseases

**Fig. 1:**
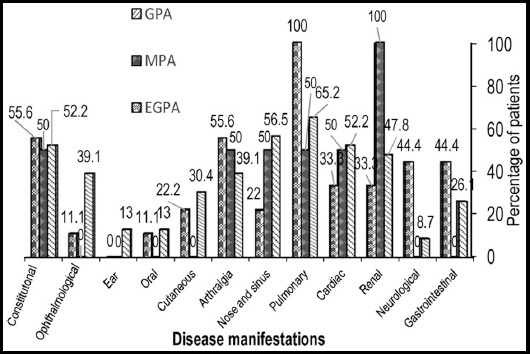
Comparison of organ system involvement in three different ANCA associated vasculitic diseases. **ANCA-** Anti-neutrophil cytoplasmic antibodies, **GPA -** Granulomatosis with polyangiitis, **MPA-** Microscopic polyangiitis, **EGPA –** Eosinophilic granulomatosis with polyangiitis

When the three AAV diseases GPA, MPA and EGPA were compared (Table-I, Fig.1) we found that, the most frequently affected system in GPA and EGPA was pulmonary (65.2%, 100% respectively) while it was renal in MPA patients (100%). Except for fever, which was found significantly more common in our GPA patients (p=0.030), all other constitutional symptoms occurred almost with similar rates in the three AAV diseases. Ophthalmological involvement was more frequent in GPA (39.1%) compared to MPA and EGPA. Ear involvement was only seen in GPA patients. Upper airways (nose and sinus) were more affected in GPA patients (56.5%). None of our MPA patients had eye, ear, oral, cutaneous, neurological or gastrointestinal manifestations. Among cutaneous symptoms, the type of rash more commonly manifested was palpable purpuric mostly observed in GPA patients. Bronchial asthma was a feature of all EGPA patients and a few GPA patients (p=0.001). Neurological involvement was significantly highest in EGPA cases (44.4%) compared to GPA and MPA patients (8.7% and 0% respectively; p=0.046).

### Hematological and immunological features of whole AAV group

None of our AAV patients had leukopenia or thrombocytopenia. However, leukocytosis (35.3%), anemia (41.2%) and thrombocytosis (23.5%) were observed (Table-II). ANCA were detected in 27 of 34 (79.4%) AAV patients and seven patients were ANCA negative. c-ANCA were detected in 21 (77.8%) and p-ANCA in 6 ANCA positive AAV patients (22.2%) and specificity of ANCA was PR3 directed in 15 (83.3%) and MPO directed in 3 (16.7%) of the 18 ANCA positive patients tested for PR3 / MPO specificity.

**Table-II:** Lab parameters in ANCA associated vasculitis patients (n=34).

Parameter	GPA n=23 N(%)	MPA n=2 N(%)	EGPA n=9 N(%)	All AAV n=34 N(%)
Leukocytosis	8(34.8)	1(50.0)	3(33.3)	12(35.3)
Anemia	11(47.8)	1(50.0)	2(22.2)	14(41.2)
Thrombocytosis	7(30.4)	0(0)	1(11.1)	8(23.5)
Eosinophilia	0(0)	0(0)	1(11.1)	1(2.9)
Prolonged ESR	13(56.5)	0(0)	4(44.4)	17(50.0)
Elevated serum creatinine	7(30.4)	2(100.0)	1(11.1)	10(29.4)
RF positive	8(34.8)	0(0)	1(11.1)	9(26.5)
Abnormal creatinine clearance	6(26.1)	2(100.0)	2(22.2)	10(29.4)
24 hour urine protein >0.5 g	5(21.7)	2(100.0)	3(33.3)	10(29.4)
ANCA Positive*	21(91.3)	2(100.0)	4(44.4)	27(79.4)
ANCA Negative	2(8.7) n=21	0(0) n=2	5(55.6) n=4	7(20.6) n=27
c-ANCA positive *	17(80.9)	1(50.0)	3(75.0)	21(77.8)
p- ANCA positive*	4(19.4)	1(50.0)	1(25.0)	6(22.2)
ANCA PR3 / MPO*	n=15 (ND=6)	n=1 (ND=1)	n=2 (ND=2)	n=18 (ND=9)
PR3 Positive	14(93.3)	1(100.0)	0(0)	15(83.3)
MPO Positive	1(6.7)	0(0)	2(100.0)	3(16.7)

ANCA: Anti-neutrophil cytoplasmic antibodies, AAV - ANCA associated vasculitis, GPA: Granulomatosis with polyangiitis, MPA: Microscopic polyangiitis, EGPA: Eosinophilic granulomatosis with polyangiitis, ESR: Erythrocyte sedimentation rate, RF: Rheumatoid factor, ND: Not done,

* p <0.05 (significant).

### Hematological and immunological features compared in three AAV diseases

Anemia and thrombocytosis were more pronounced in GPA compared to other AAV while eosinophilia was observed in EGPA patients only (Table-II). Renal abnormalities were more frequently noted in MPA patients. ANCA were positive in 91.3% GPA cases (21 of 23 cases), all MPA cases (both the cases), compared to only 44.4% EGPA (4 of 9 cases) patients (p=0.010). In GPA, c-ANCA were more frequent, detected in 17 (80.9%) compared to p-ANCA detected in 4 (19.1%) of the 21 ANCA positive patients (p=0.018). We also found c-ANCA in 1 of the 2 MPA (50%) cases and in three of the four (75%) EGPA ANCA positive cases. ANCA were PR3 directed in 93.3% of GPA cases tested for PR3/MPO. We found that ANCA were MPO directed in 1 (6.7%) of our GPA patients who was positive for p-ANCA. The c-ANCA positive MPA case tested for PR3/ MPO was found PR3 positive and MPO negative. Both the cases of EGPA tested for PR3/MPO were PR3 negative and MPO positive, one of them was c-ANCA positive and the other one was p-ANCA positive.

### Association of disease manifestations and morbidity with p/c ANCA and PR3 / MPO ANCA in AAV group

We didn't find relation between p- or c- ANCA and disease manifestations in our AAV patients except for the association of neurological symptoms with p-ANCA (p=0.039). Of the 21 c-ANCA positive patients in the whole AAV group, 8 (38.1%) were hospitalized for disease flares or infections compared to 4 (66.7%) of the 6 p-ANCA positive cases (Table-II), showing association of p-ANCA with morbidity compared to c-ANCA (p<0.05). Similarly MPO positivity was found more associated with morbidity than PR3 positivity (p<0.05). Two (66.7%) of the 3 MPO positive cases (in the whole AAV group) were hospitalized, had infections and disease flares compared to 5 (33.3%) of the 15 PR3 positive cases.

### Therapy, morbidity and outcome of AAV group

**Fig. 2:**
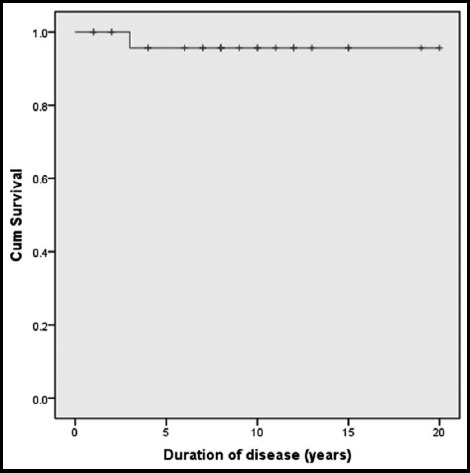
Kaplan - Meir Survival curve of AAV patients.

All AAV patients were treated with corticosteroids (Table-III). Twenty patients (58.8%) were treated with a combination of corticosteroids and IV CYC. IV methyl prednisolone and azathioprine were individually received by 17 (50.0%) patients each. Plasmapheresis was administered to one of the MPA patients and 4 GPA patients (11.8%) received rituximab doses. Complications included infections, cushings syndrome, aseptic necrosis of hip and shoulder, complex pulmonary aspergilloma, mastoiditis, side effect dysnea and itching of hands and feet due to rituximab. Sixteen (47.1%) patients were hospitalized for various reasons including disease flares and infections. Ten (29.4%) AAV patients had episodes of infections, namely pneumonia in four patients, septicemia in four, esophageal candidiasis and bacterial meningitis in one and herpes zoster in one patient. Five (14.7%) AAV patients were admitted to ICU. All patients were in remission on treatment at follow up. One elderly EGPA patient died from infection and multi-organ failure. Survival analysis of AAV patients by Kaplan-Meier survival analysis yielded both 5-year and 10-year survival rates of 95% (Fig.2).

**Table-III:** Therapy in ANCA associated vasculitis patients (n=34).

Therapy	N (%)
Prednisolone	34(100.0)
Methyl prednisolone	17(50.0)
Oral cyclophosphamide	6(17.6)
IV cyclophosphamide	20(58.8)
Azathioprine	17(50.0)
Mycophenolate mofetil	3(8.8)
Methotrexate	5(14.7)
Infliximab	1(2.9)
Rituximab	4(11.8)
Plasmapheresis	1(2.9)

ANCA: Anti-neutrophil cytoplasmic antibodies.

## DISCUSSION

In this study we are reporting on our experiences with AAV in patients from our region. Studies reporting on geographic variations in the frequencies of different AAV have shown that GPA is more prevalent than MPA in northern Europe and Germany whereas MPA is much more common than GPA in southern Europe. In Japan all patients were found to have MPA with MPO positive ANCA while GPA or PR3 ANCA associated vasculitis was not observed. In China there is predominance of MPA while GPA is rare and it is noteworthy that GPA patients from China are mostly p-ANCA / MPO-ANCA positive while in Europe, GPA is mostly c-ANCA / PR3 -ANCA positive.^12^-^14^ In northwestern Turkey frequency of AAV was similar to that in southern Europe.^15^ In our patients GPA was more frequent than MPA and EGPA and c-ANCA / PR3 ANCA associated vasculitis was more common than p-ANCA / MPO-ANCA similar to European patients. These geographical differences suggest the role of genetic factors in the pathogenesis of AAV.

AAV is known to affect men slightly more frequently than women which is in accordance with our finding.^13^,^16^ Disease manifestations are known to occur with variable frequencies in GPA, MPA and EGPA patients.^4^,^16^ Among constitutional symptoms we found that GPA patients present with fever more commonly than MPA and EGPA patients similar to previous studies.^17^ Eye involvement has been reported in 60% GPA, 30% MPA and 10% EGPA patients.^4^ We found eye involvement by GPA in 39.1% and by EGPA in 11.1% patients and in none of our MPA patients. The most common cutaneous lesion in our patients was palpable purpura similar to previous studies.^7^,^8^ GPA manifests with classic triad of involvement of upper respiratory tract, lungs and kidneys.^16^,^18^ As per previous reports nose and sinus symptoms are seen in 90% of GPA patients, 30% of MPA and 80% of EGPA patients.^4^ In our patients nose and sinus involvement was observed in 56.5% of GPA, 50% of MPA and 22% of EGPA patients. Pulmonary involvement has been shown to occur with the rates of 80% in GPA, 60% in MPA and 100% in EGPA patients which are comparable to our rates of 65.2% in GPA, 50% in MPA and 100% in EGPA patients.^4^ All our EGPA patients had asthma as has been previously reported.^4^,^16^ Renal involvement in our patients is similar to the rates reported elsewhere but in GPA patients we had a lower percentage of cases (47.8%). As per literature, kidneys are more involved in MPA affecting almost 100% of patients while 80% of GPA and 40% of EGPA patients are known to manifest renal symptoms.^4^,^17^ Peripheral neuropathy has been reported to manifest predominantly in EGPA patients observed in 76% of patients compared to GPA and MPA (51% and 37% respectively).^17^ Consistent with this finding, we found that our EGPA patients were more likely to have neurological manifestations (mononeuritis multiplex and sensor motor-polyneuropathy) (44.4%) compared to GPA and MPA patients (8.7% and 0% respectively).

Leukocytosis as high as 92% has been reported in AAV patients while in our study, it was observed in 35.3% of patients.^17^ ANCA were more frequently detected in our GPA and least frequently in EGPA patients as in previous studies which reported 83.6-90% ANCA in GPA compared to 70-78.5% in MPA and 24-45% in EGPA patients which is similar to our finding of 91.3% positive ANCA in GPA compared to 44.4% in EGPA patients.^4^,^17^ As per literature c-ANCA are more prevalent than p-ANCA in GPA (90% c- vs 10% p-ANCA) and p-ANCA are more prevalent than c-ANCA in MPA (80% p- vs 10% c-ANCA) and EGPA (75% p- vs 10% c-ANCA).^4^,^7^,^16^ We found predominance of c-ANCA in our GPA patients, however we also found higher rate of c-ANCA compared to p-ANCA in MPA and EGPA cases; c-ANCA were detected in one of the two MPA (50%) cases and in three of the four EGPA (75%) ANCA positive cases. ANCA were PR3 directed in 93.3% of GPA cases tested for PR3/ MPO specificity, consistent with previous reports which showed that ANCA is mostly PR3 directed in GPA.^4^,^7^,^16^

Use of high dose corticosteroids and IV CYC for induction and azathioprine or methotrexate with steroid tapering for maintenance improved the outcome of our AAV patients.^10^,^19^,^20^ Four of our GPA cases required rituximab therapy to control their symptoms which has been shown to be effective in relapsing and refractory AAV.^20^,^21^ Since the introduction of treatment regimen involving IV CYC for induction therapy with glucocorticoids and azathioprine or methotrexate for maintenance therapy, five year survival has exceeded 80%.^4^ However, mortality occurs due to alveolar hemorrhage, renal failure and infections, mostly in older patients. Some studies have reported survival rates between 45% and 91% at 5 years and 65.3% and 88% at 10 years.^11^,^15^,^22^ Our patients showed a 5-year and 10-year survival rate of 95% each. Our improved survival may be due to the use of intensive therapeutic modalities including IV CYC and rituximab.

## CONCLUSION

To our knowledge, ours is the first study describing the clinical and immunological features, therapy, outcome and survival of AAV patients from Saudi Arabia. Disease manifestations in our AAV patients were similar to AAV patients from other countries. ANCA pattern and specificity were similar to Caucasian AAV patients but different from Japanese and Chinese AAV patients. We report better survival rates in our AAV patients. As AAV are challenging to diagnose due to protean disease manifestations and as refractory forms are difficult to treat, better diagnostic and therapeutic approach is mandatory for improved outcome.

### Authors Contribution

**ASA:** Conceived, designed and supervised the study, did literature search, manuscript preparation and final approval of manuscript.

**NK:** Did data collection, statistical analysis, literature search, manuscript preparation.
